# Preliminary safety and imaging efficacy of the near-infrared fluorescent contrast agent DA364 during fluorescence-guided surgery in dogs with spontaneous superficial tumors

**DOI:** 10.18632/oncotarget.27633

**Published:** 2020-06-16

**Authors:** Sophie Favril, Chiara Brioschi, Katrien Vanderperren, Eline Abma, Emmelie Stock, Nausikaa Devriendt, Ingeborgh Polis, Hilde De Cock, Alessia Cordaro, Luigi Miragoli, Paolo Oliva, Giovanni Valbusa, Charline Alleaume, Isabelle Tardy, Alessandro Maiocchi, Fabio Tedoldi, Francesco Blasi, Hilde de Rooster

**Affiliations:** ^1^ Small Animal Department, Faculty of Veterinary Medicine, Ghent University, Merelbeke, Belgium; ^2^ Cancer Research Institute Ghent, Ghent, Belgium; ^3^ Bracco Imaging SpA, c/o BioIndustry Park, Colleretto Giacosa, Italy; ^4^ Department of Veterinary Medical Imaging and Small Animal Orthopaedics, Faculty of Veterinary Medicine, Ghent University, Merelbeke, Belgium; ^5^ Medvet/Algemeen Medisch Laboratorium, Antwerpen, Belgium; ^6^ Ephoran Multi-Imaging Solutions, Colleretto Giacosa, Italy; ^7^ Bracco Suisse S.A., Plan-les-Ouates, Switzerland

**Keywords:** fluorescence-guided surgery, fluorescent contrast agent, near-infrared fluorescence imaging, spontaneous tumors, canine

## Abstract

Tumor-targeting contrast agents may facilitate resection of solid neoplasms during fluorescence-guided surgery. Preliminary safety and imaging efficacy of the near-infrared fluorescent probe DA364 were evaluated during surgical resection of spontaneous solid tumors in 24 dogs. Intra-operative imaging was performed *in situ* and on excised specimens to evaluate fluorescence intensities of tumor and adjacent tissues. After standard-of-care tumor resection, the wound bed was imaged again, and additional tissue was excised if residual fluorescence was detected.

DA364 was well tolerated after intravenous administration. The median tumor-to-background ratio *in situ* for mammary tumors, mast cell tumors and sarcomas was 1.8 (range 1.2–3.9), 2.2 (range 1.0–5.6), and 4.2 (range 2.0–4.3), respectively. Qualitative intra-operative tumor identification was feasible in half of the cases. Remaining fluorescence was detected in four wound beds that contained residual disease, and in11 tumor-free wound beds, confirmed by histopathology.

Overall, DA364 did not raise safety concerns and showed accumulation in different types of spontaneous tumors, showing potential to pinpoint residual disease. Larger clinical trials are necessary to select accurate dosing and imaging protocols for specific indications to evaluate the sensitivity and specificity of the agent.

## INTRODUCTION

Despite many recent improvements in the medical treatment of cancer, surgery remains the most effective therapeutic strategy for the majority of patients with solid tumors [1]. Tissue palpation, visual inspection, and real-time frozen section analyses are commonly used by surgeons to identify tumor margins during resection, and to assess the presence of residual disease [1]. However, positive tumor margins after surgical resection are a common histopathological finding [2–4], which strongly increases the risk of cancer recurrence [5]. Thus, there is a high need for the implementation of new intra-operative methodologies that can provide accurate real-time tumor margin assessment to overcome the limitations of standard-of-care practice.

Fluorescence imaging is a safe and sensitive technique that may assist the surgeon by real-time delineation of tumor lesions during resection through visualization of specific fluorescent probes [6–8]. Particularly intra-operative near-infrared fluorescence (NIRF; 700–900 nm) imaging is currently being investigated using contrast agents that preferentially accumulate in tumor tissues, and that may be detected at greater depths and with higher image resolution than tracers emitting in the visible spectrum [8]. Indocyanine Green (ICG), the only NIRF agent currently approved for human use (*i. e*., angiography, lymph node (LN) mapping, liver function testing), has demonstrated limited sensitivity and specificity for intra-operative tumor identification in human and veterinary trials [9–11], due to its unspecific tumor accumulation mechanism. Novel contrast agents that recognize molecular epitopes overexpressed by tumor tissues (*i. e.,* vascular endothelial growth factor, folate receptor, carcinoembryonic antigen) have shown safety and efficacy during clinical trials and hold great promise to be implemented in clinical practice [12–16].

In this study, preliminary safety and imaging efficacy of the NIRF agent DA364 were evaluated during resection of superficial solid tumors in canine patients. DA364 is composed of Cy5.5, a water-soluble cyanine dye (fluorescence quantum yield in serum: Cy5.5, 21%; ICG, 9%) [17, 18], and a cyclic Arg-Gly-Asp (RGD) peptidomimetic moiety, targeting integrins, which are the main cellular adhesion protein family implicated in nearly every step of tumor progression [19, 20]. It has previously been reported that DA364 has high affinity and specificity for integrin α_v_β_3_ *in vitro*, and excellent imaging performance *in vivo* to detect tumor masses in rodent models of human cancers [21, 22]. *In vitro* evaluations were conducted to test the biological properties of the DA364 batch used for the canine trial. Based on the promising pre-clinical results, we hypothesize that DA364 accumulates in spontaneous tumors in dogs after intravenous (IV) administration, allowing intra-operative tumor detection and eradication of residual disease in the wound bed. Secondary objectives of the trial are: 1) to evaluate the probe’s tissue kinetics after injection, 2) to determine the optimal dose and time interval for tumor detection during surgery, and 3) to assess the pattern of integrin receptor expression in different tissues.

## RESULTS

### *In vitro* analyses

**Figure 1:**
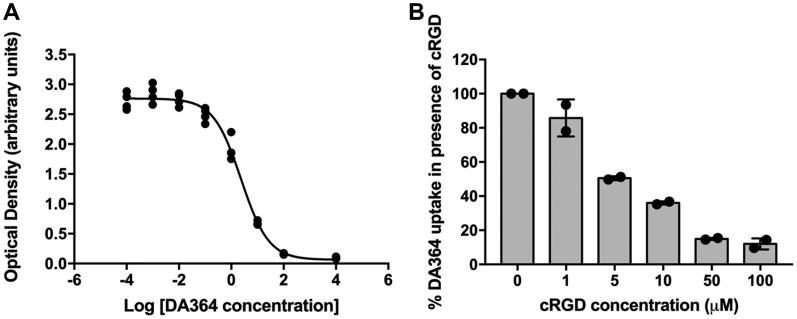
*In vitro* analyses. (**A**) Representative sigmoid curve revealing single-digit nanomolar affinity of DA364 for the human α_v_β_3_ integrin receptor. (**B**) Dose-response inhibition of DA364 uptake in human melanoma WM266 cells by the unconjugated cRGD vector.

DA364 affinity to human α_v_β_3_ integrin was evaluated in a solid phase binding assay via displacement of vitronectin, the physiological ligand of the receptor (Figure 1A). DA364 was able to compete for the binding to the target with single-digit nanomolar potency (IC_50_ 2.5 ± 0.2 nM), confirming the findings from previous batches of the product [21].

DA364 showed efficient internalization in human melanoma WM266 cells which overexpress the integrin receptor α_v_β_3_ [23] (Figure 1B). The treatment with the competitor inhibited DA364 internalization in a dose-dependent manner. An uptake inhibition of 85% was reached with a 50-fold concentration of the unconjugated cRGD peptidomimetic moiety.

### Patient characteristics and study workflow

Between February 2016 and April 2017, 24 dogs with 32 suspected superficial solid tumors were included in the clinical trial. Table 1 illustrates the general characteristics of the dogs, the tumor type (s) and respective trial data. The histopathological diagnosis of the major tumors revealed the presence of mast cell tumor (*n* = 9), mammary gland adenocarcinoma (*n* = 6), mammary gland adenoma (*n* = 2), vaginal leiomyosarcoma (*n* = 1), soft tissue sarcoma (*n* = 1), osteosarcoma (*n* = 1), cutaneous melanoma (*n* = 1), adenocarcinoma of the apocrine gland (*n* = 1), cysts of the mammary gland (*n* = 1), and lipoma (*n* = 1). Histopathological diagnosis of second smaller tumors that were identified during physical examination in eight dogs revealed the presence of an additional mammary gland adenoma (*n* = 5), mammary gland adenocarcinoma (*n* = 2), and pyogranulomatous dermatitis (*n* = 1). The cysts of the mammary gland and the pyogranulomatous dermatitis lesion were not included in any further analyses since both lesions do not have a neoplastic origin.

**Table 1:** General patient and tumor characteristics and respective trial data of dogs enrolled for fluorescence imaging

Dog	TI (h)	Dose (mg/m^2^)	Breed (gender)	Age (y)	Weight (kg)	BCS [31]	Tumor/lesion characteristics			
							***Type***	***Stage [32]***	***Grade***	***Site***
**1**	24	0.06	American Staffordshire Terrier (F)	6.7	27.0	4	1a: Adenoma	T1N0M0	—	Mammary gland
							1b: Adenoma	T1N0M0	—	
**2**	24	0.6	Jack Russell Terrier (Fn)	12.3	4.5	4	2a: Adenocarcinoma	T2N1M0	III	Mammary gland
							2b: Adenocarcinoma	T1N0M0	I	
**3**	24	1.8	Weimaraner (F)	6.3	40.0	6	Cysts	—	—	Mammary gland
**4**	24	1.8	Malinois (Fn)	14.0	27.0	4	4a: Adenocarcinoma	T3N1M0	III	Mammary gland
							4b: Adenoma	T1N0M0	—	
**5**	24	1.8	Yorkshire Terrier (F)	13.9	3.5	3	5a: Leiomyosarcoma	T3N0M0	—	Vagina Mammary gland
							5b: Adenoma	T1N0M0	—	
**6**	24	1.8	American Staffordshire Terrier (Fn)	10.0	23.0	4	Mast cell tumor	Ia	I	Mid-femur
**7**	24	1.8	Labrador retriever (Fn)	8.2	34.5	5	Mast cell tumor	Ia	II	Ventral abdomen
**8**	24	1.8	Cross breed (M)	12.9	5.5	4	Mast cell tumor	Ia	II	Ventral abdomen
**9**	24	1.8	Maltese (Mn)	8.1	8.5	4	Mast cell tumor	Ia	II	Left femur
**10**	24	1.8	Jack Russell Terrier (F)	5.7	8.5	6	Mast cell tumor	IIb	III	Left thoracic wall
**11**	24	1.8	Labrador retriever (F)	7.5	31.5	5	Mast cell tumor	Ia	II	Tarsal region
**12**	24	3.0	Maltese (F)	6.3	6.5	4	Adenoma	T1N0M0	—	Mammary gland
**13**	24	3.0	Cross breed (F)	9.3	12.0	4	13a: SCC/Adenocarcinoma	T2N0M0	I	Mammary gland
							13b: Adenoma	T1N0M0		
**14**	24	1.8	German shepherd (Mn)	3.9	40.0	5	Soft tissue sarcoma	T3N0M0	II	Elbow region
**15**	24	1.8	Dobermann (Mn)	1.7	39.0	4	Osteosarcoma	T2N0M0	—	Left thoracic wall
**16**	24	1.8	Shar-Pei (M)	12.4	24.0	5	Melanoma	T2N0M0	High grade	Left cheeck
**17**	24	1.8	Border Collie (M)	10.3	26.0	6	Adenocarcinoma	T3N0M0	—	Apocrine glands surrounding the left anal sac
**18**	24	1.8	Labrador retriever (Fn)	9.5	41.0	6	Lipoma	—	—	Right inguinal area
**19**	48	1.8	Miniature Poodle (F)	8.4	4.0	4	19a: Adenocarcinoma	T1N0M0	I	Mammary gland
							19b: Adenoma	T1N0M0	—	
**20**	48	1.8	Jack Russell Terrier (F)	12.8	7.0	5	20a: Adenocarcinoma	T1N0M0	I	Mammary gland
							20b: Adenocarcinoma	T1N0M0	I	
**21**	48	1.8	Labrador retriever (F)	9.9	37.0	5	Adenocarcinoma	T2N0M0	I	Mammary gland
**22**	48	1.8	Labrador retriever (M)	11.1	41.0	6	22a: Mast cell tumor	Ia	III	Right tarsus
							22b: PGD	—	—	
**23**	48	1.8	Weimaraner (Mn)	6.1	39.0	6	Mast cell tumor	Ia	II	Left stifle
**24**	48	1.8	American Staffordshire Terrier (Mn)	10.3	35.0	5	Mast cell tumor	IIa	III	Right tarsus

Abbreviations: BCS, body condition score; F, female; Fn, female neutered; M, male; Mn, male neutered; PGD, pyogranulomatous dermatitis; SCC, squamous cell carcinoma; TI, time interval.

**Figure 2:**
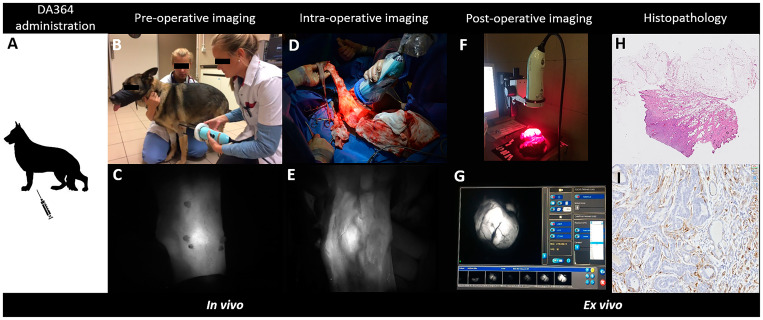
Overview of the procedure to test DA364 as fluorescent contrast agent to highlight tumors during surgery. The workflow consists of the intravenous administration of DA364 24 h or 48 h prior to surgery (**A**), followed by pre-operative imaging (**B**, **C**: procedure and fluorescent image in panel respectively), intra-operative imaging (**D**, **E**: procedure and correspondent fluorescent image in panel respectively) and post-operative imaging (**F**, **G**: procedure and correspondent fluorescent image, visualized on screen, respectively). Excised tissues were sliced in 4 μm-thick sections and underwent tumor assessment by Hematoxylin and Eosin histopathology (**H**), and evaluation of the β_3_ integrin by immunohistochemistry (**I**).

The study workflow (Figure 2) started with the IV administration of DA364 at various doses either 24 or 48 h prior to surgery, followed by pre-operative imaging to assess tissue kinetics, then intra-operative imaging to evaluate fluorescence in tumors and surroundings, and post-operative imaging of resected specimen to verify the distribution of the fluorescent signal. Furthermore, the excised specimens were prepared for histopathological assessment and for proteomic analyses to evaluate the expression of the integrin receptors.

### Pre-operative imaging

No adverse effects were observed after IV administration of DA364. All patients were clinically stable; heart rate, respiratory rate and systolic blood pressure were within physiological ranges throughout the experiment. Reliable pre-operative kinetics were obtained in 12 tumors (5a, 6, 7, 9, 11, 14, 17, 18, 19a, 20a, 23, 24). The selection of sufficient transcutaneous images of tumors was based on optimal image quality, which was highly affected by parameters such as the thickness and color of the skin, and transcutaneous fluorescence imaging feasibility, which was affected by the location of tumor. Analysis of the signal decay over time revealed a peak (T_max_) at 1.6 h post-injection (PI) and long-lasting tissue retention in the tumors (T_1/2_: 16.5 h).

### Intra-operative imaging

The tumor was always removed with a certain margin of surrounding tissue, depending on the current recommendations for each tumor type, meaning no attempt was done to resect the tumor based on the intensity of the fluorescent signal. The dose of 0.06 mg/m^2^ was too low for intra-operative NIRF tumor detection. The dose of 0.6 mg/m^2^ was sufficient to detect the tumor *in situ*, but the dose of 1.8 mg/m^2^ revealed sharper images, and therefore was selected as primary dose for the trial (Table 1). The dose of 3 mg/m^2^ did not improve image quality compared to the 1.8 mg/m^2^ dose whereas increased background fluorescence was observed.

**Figure 3:**
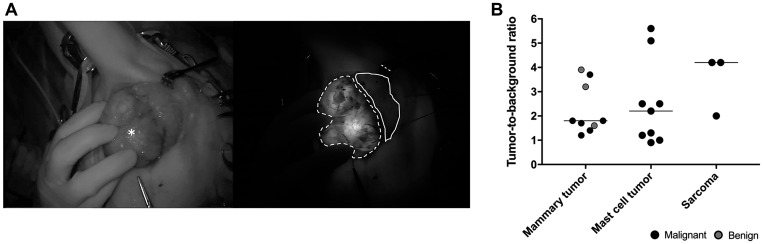
Intra-operative imaging. (**A**) Representative brightfield (left) and fluorescence (right, 40 ms exposure) images *in situ* of an adenocarcinoma 24 h after administration of DA364 (patient 17; 1.8 mg/m^2^). The asterisk denotes the mass, the dotted line denotes the tumor region of interest and the solid line denotes the background region of interest. (**B**) Evaluation of tumor-to-background ratios in different tumor types. Horizonal bars represent medians.

The median tumor-to-background ratio (TBR) *in situ* for all mammary tumors, mast cell tumors and sarcomas was 1.8 (range 1.2–3.9), 2.2 (range 1.0–5.6), and 4.2 (range 2.0–4.3), respectively (Table 2, Figure 3). The analysis was not performed on six mammary tumors (two of which were adenocarcinomas) because the image quality was suboptimal, either due to the low dose of contrast administered or due to a tumor diameter of less than 5 mm. Although the intra-operative TBR was ≥ 1.0 in all tumors (with the exception of the highly pigmented melanoma), the intra-operative qualitative evaluation showed a distinctive positive signal of the exposed lesion compared to adjacent tissues in only 15 out of 30 tumors (Table 2). The lowest TBR in the tumor that was qualitatively identified was 1.2. Furthermore, there was no significant difference in TBRs between malignant tumors and benign lesions. Malignant mammary gland tumor patients imaged 48 h after administration showed subjectively lower background fluorescence than patients imaged after 24 h. However, delaying the surgery and acquisition to 48 h PI did not result in appreciable improvement of the *in vivo* TBR.

**Table 2:** Evaluation of fluorescence intensity *in situ* and on excised specimens

Dog	Tumor type	Dose (mg/m^2^)	Intra-operative (*in vivo*)				Post-operative (*ex vivo*)		
			*Lesion*	*Background*	*TBR*	*Qualitative evaluation^a^*	*Center*	*Margin*	*TMR*
**1**	1a: Adenoma	0.06	NA^b^	NA^b^	**NA^b^**	—	NA^b^	NA^b^	**NA^b^**
	1b: Adenoma		NA^b^	NA^b^	**NA^b^**	—	NA^b^	NA^b^	**NA^b^**
**2**	2a: Adenocarcinoma	0.6	40.7	22.3	**1.8**	—	144.1	79.0	**1.8**
	2b: Adenocarcinoma		NA^b^	NA^b^	**NA^b^**	—	NA^b^	NA^b^	**NA^b^**
**4**	4a: Adenocarcinoma	1.8	141.9	82.6	**1.7**	+	51.8	11.6	**4.5**
	4b: Adenoma		172.8	107.1	**1.6**	+	102.6	30.6	**3.4**
**5**	5a: Leiomyosarcoma	1.8	49.2	24.4	**2.0**	—	NA^c^	NA^c^	**NA^c^**
	5b: Adenoma		115.9	36.7	**3.2**	+	100.9	52.5	**1.9**
**6**	Mast cell tumor	1.8	79.7	15.6	**5.1**	+	74.8	9.2	**8.1**
**7**	Mast cell tumor	1.8	35.4	6.3	**5.6**	+	51.3	25.9	**2.0**
**8**	Mast cell tumor	1.8	63.3	63.6	**1.0**	—	74.2	6.1	**12.2**
**9**	Mast cell tumor	1.8	24.4	11.1	**2.2**	+	57.0	26.2	**2.2**
**10**	Mast cell tumor	1.8	60.5	63.3	**1.0**	—	NA^d^	NA^d^	**NA^d^**
**11**	Mast cell tumor	1.8	39.1	33.2	**1.2**	+	39.6	15.7	**2.5**
**12**	Adenoma	3.0	75.2	19.4	**3.9**	—	148.5	91.7	**1.6**
**13**	13a: SCC /Adenocarcinoma	3.0	28.9	20.7	**1.4**	—	50.0	11.4	**4.4**
	13b: Adenoma		NA^d^	NA^d^	**NA^d^**	—	NA^d^	NA^d^	**NA^d^**
**14**	Soft tissue sarcoma	1.8	130.5	31.4	**4.2**	+	121.9	22.6	**5.4**
**15**	Osteosarcoma	1.8	96.4	22.7	**4.3**	+	51.7	43.2	**1.2**
**16**	Melanoma	1.8	13.0	78.8	**0.2**	—	1.1	19.8	**0.1**
**17**	Adenocarcinoma	1.8	99.8	31.4	**3.2**	+	142.8	9.9	**14.4**
**18**	Lipoma	1.8	61.7	14.4	**4.3**	+	78.5	27.9	**2.8**
**19^e^**	19a: Adenocarcinoma	1.8	113.0	30.2	**3.7**	+	166.2	26.1	**6.4**
	19b: Adenoma		NA^d^	NA^d^	**NA^d^**	—	NA^d^	NA^d^	**NA^d^**
**20^e^**	20a: Adenocarcinoma	1.8	51.6	28.7	**1.8**	—	57.6	29.1	**2.0**
	20b: Adenocarcinoma		NA^d^	NA^d^	**NA^d^**	+	NA^d^	NA^d^	**NA^d^**
**21^e^**	Adenocarcinoma	1.8	20.6	16.6	**1.2**	—	61.2	18.0	**3.4**
**22^e^**	Mast cell tumor	1.8	38.1	15.0	**2.5**	+	69.1	25.9	**2.7**
**23^e^**	Mast cell tumor	1.8	42.9	17.0	**2.5**	—	38.6	10.9	**3.5**
**24^e^**	Mast cell tumor	1.8	74.9	57.5	**1.3**	+	45.9	20.0	**2.3**

Fluorescence intensity is expressed as mean value of the region of interest. ^a^Qualitative evaluation indicates whether differentiation of the tumor was feasible during surgical resection of the tumor. ^b^Measurement of the fluorescent signal was not feasible because the administered dose was too low. ^c^Measurement of the *ex vivo* fluorescent signal was not feasible because no background region of interest could be selected since the resected specimen completely consisted of vaginal tumor. ^d^Measurement of the fluorescent signal was not performed because the tumor diameter was less than 5 mm. ^e^Dogs that received the probe 48 h prior to surgery. Abbreviations: NA, not available; SCC, squamous cell carcinoma; TBR, tumor-to-background ratio; TMR, tumor-to-margin ratio.

Complete resection of the tumor was deemed successful by standard-of-care evaluation in 27 out of 30 lumps, and histopathology claimed negative surgical margins in 26 of those (Table 3). In two mast cell tumor cases (patients 11 and 22) and in the melanoma (patient 16), complete resection was not feasible due to the location (proximity of the eye and tarsus). As expected, tumor margins contained microscopic tumor remnants and fluorescence was detected in the wound bed (true positives). In one mast cell tumor (patient 10), a fluorescence signal was detected in the surgical bed, and histopathology showed that the tumor tissue reached until the section margins (true positive, undetected during surgery by standard-of-care examination). No remaining fluorescence was noted in 15 of the 26 histopathologically-clean wound beds (true negatives, mostly mammary gland tumors). In the 11 remaining wound beds, fluorescence was noted but the biopsies did not contain tumor cells (false positives, mostly mast cell tumors and sarcomas) (Table 3).

**Table 3:** Intra-operative evaluation of the lesion by standard-of-care inspection and fluorescence in the wound bed

Tumor	Lump margin inspection (standard-of-care)^a^		Wound bed assessment (fluorescence imaging)^b^		
	*Surgeon*	*Histopathology^c^*	*Fluorescence in wound bed (samples)*	*Histopathology^c^*	*Description*
1a: Adenoma	Negative	Negative	Negative	—	—
1b: Adenoma	Negative	Negative	Negative	—	—
2a: Adenocarcinoma	Negative	Negative	Negative	—	—
2b: Adenocarcinoma	Negative	Negative	Negative	—	—
4a: Adenocarcinoma	Negative	Negative	Negative	—	—
4b: Adenoma	Negative	Negative	Negative	—	—
5a: Leiomyosarcoma	Negative	Negative	Positive (1)	Negative	Healthy vaginal wall
5b: Adenoma	Negative	Negative	Negative	—	—
6: Mast cell tumor	Negative	Negative	Positive (1)^d^	Negative	Skin with minimal perivascular lymphocytic infiltrate
7: Mast cell tumor	Negative	Negative	Positive (1)	Negative	Adipose and connective tissue
8: Mast cell tumor	Negative	Negative	Positive (1)	Negative	Adipose tissue and skeletal muscle
9: Mast cell tumor	Negative	Negative	Negative	—	—
10: Mast cell tumor	Negative	Positive	Positive	NA^e^	NA^e^
11: Mast cell tumor	Positive	Positive	Positive (1)	Negative	Adipose and connective tissue
12: Adenoma	Negative	Negative	Positive^f^	NA^f^	NA^f^
13a: SCC /Adenocarcinoma	Negative	Negative	Positive (1)	Negative	Mammary gland
13b: Adenoma	Negative	Negative	Positive^f^	NA^f^	NA^f^
14: Soft tissue sarcoma	Negative	Negative	Positive (2)	Negative	Skeletal muscle
				Negative	Normal tendon
15: Osteosarcoma	Negative	Negative	Positive (2)	Negative	Collapsed lung lobe
				Negative	Thickened pleura
16: Melanoma	Positive	Positive	Positive (1)	Negative	Mucinous connective tissue
17: Adenocarcinoma	Negative	Negative	Negative	—	—
18: Lipoma	Negative	Negative	Negative	—	—
19a: Adenocarcinoma	Negative	Negative	Negative	—	—
19b: Adenoma	Negative	Negative	Negative	—	—
20a: Adenocarcinoma	Negative	Negative	Negative	—	—
20b: Adenocarcinoma	Negative	Negative	Negative	—	—
21: Adenocarcinoma	Negative	Negative	Negative	—	—
22: Mast cell tumor	Positive	Positive	Positive	NA^e^	NA^e^
23: Mast cell tumor	Negative	Negative	Positive (1)	Negative	Normal skin
24: Mast cell tumor	Negative	Negative	Positive (1)	Negative	Subcutis with large blood vessel

^a^Positive by standard-of-care assessment denotes incomplete resections of the lump or suspicion of contamination in the wound bed. ^b^Positive by fluorescence imaging denotes the presence of a fluorescent signal in the resected sample. ^c^Positive by pathology denotes the presence of tumor tissue. ^d^False positive tissue removed adjacent to neoplastic tissue and not from the wound bed. ^e^No additional biopsy could be taken because of the anatomical location. ^f^No addititonal biopsy was taken because the high homogenous background was attributed to the high dose that these dogs received. Abbreviation: NA, not available.

### Post-operative imaging

**Figure 4:**
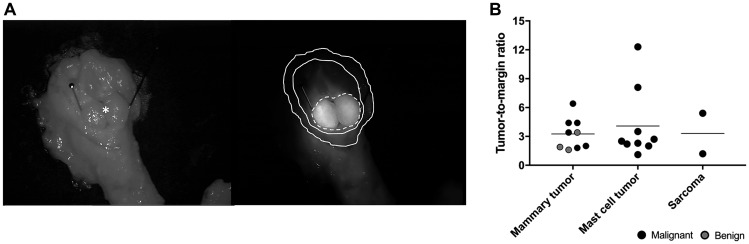
Post-operative imaging. (**A**) Representative brightfield (left) and fluorescence (right, 40 ms exposure) images of an excised mammary gland adenocarcinoma 48 h after administration of DA364 (patient 19; 1.8 mg/m^2^). The asterisk and the pins denote the mass, the dotted line denotes the tumor region of interest (ROI), the inner solid line the background ROI, and the outer solid line the margin ROI. (**B**) Evaluation of tumor-to-margin ratios in different tumor types. Horizonal bars represent medians.

The fluorescence intensity in the center (tumor) and the outer edge (surgical margin) of the excised lumps was evaluated before formalin fixation (Figure 4). Eight samples were not included due to suboptimal image quality (Table 2). The median tumor-to-margin ratio (TMR) for all benign tumors and malignant tumors (melanoma not included) was 2.4 (range 1.6–3.4) and 3.4 (range 1.2–14.4), respectively. Although subjectively a higher TMR was noticed in malignant tumors compared to benign lesions, statistical significance was not reached (*p* = 0.203). The median TMR *ex vivo* for all mammary tumors, mast cell tumors and sarcomas was 3.4 (range 1.6–6.4), 2.6 (range 2.0–12.2), and 3.3 (range 1.2–5.4) respectively.

The signal-to-background ratio (SBR) in metastatic lymph nodes (3.28 ± 0.37) was significantly higher than in normal lymph nodes (1.58 ± 0.12) (t (4.83) = –4.32; *p* = 0.008) (Table 4). Qualitative evaluation of the excised LNs revealed a positive signal in all metastatic LN, while only two non-metastatic LNs showed detectable fluorescence.

**Table 4:** Quantitative and qualitative evaluation of fluorescence in excised regional lymph nodes (LN)

Dog	Identification LN	Fluorescence LN	Fluorescence background	SBR^a^	Qualitative evaluation^b^	Histopathology^c^
1	Superficial inguinal	41.8	38.5	**1.09**	—	Negative
2	Superficial inguinal	12.3	4.4	**2.80**	+	Positive
3	Superficial inguinal	24.6	16.2	**1.52**	—	Negative
4	Superficial inguinal	21.6	6.0	**3.60**	+	Positive
10	Axillary	26.1	6.2	**4.21**	++	Positive
12	Superficial inguinal	59.3	39.6	**1.50**	—	Negative
13	Superficial inguinal	17.5	8.1	**2.16**	+	Negative
14	Axillary	12.8	8.3	**1.54**	—	Negative
19	Superficial inguinal	21.7	13.9	**1.56**	+	Negative
21	Superficial inguinal	7.2	4.2	**1.71**	—	Negative
22	Popliteal	30.5	8.2	**3.72**	+	Positive
24	Popliteal	36.7	17.6	**2.09**	+	Positive

^a^Signal-to-background ratios obtained in *ex vivo* LN cut in half before formalin fixation at 40 ms exposure vs. adjacent fat tissue (background). ^b^Qualitative evaluation denotes whether detection of a fluorescent signal was feasible in the LNs *ex vivo* before fixation. ^c^Histopathological analysis confirmed the presence (positive) or absence (negative) of neoplastic cells in the LN. Abbreviation: SBR, signal-to-background ratio.

### Western blot and immunohistochemistry

**Figure 5:**
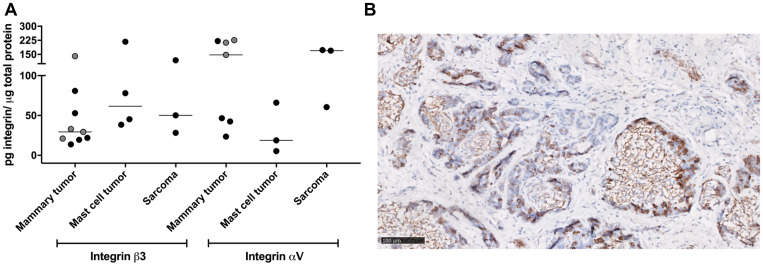
*Ex vivo* evaluation of integrins expression. (**A**) Western blot analysis of integrin receptor subunits expression per tumor type. Black circles, malignant tumors; grey circles, benign tumors. Horizonal bars represent medians. (**B**) Representative image of β_3_ integrin immunostaining on a mammary gland adenocarcinoma showing tumor cell and stromal staining (brown, β_3_ integrin positive stain; cell nuclei are stained with Hematoxylin in blue). Scale bar, 100 microns.

The expression of the integrin subunits α_V_ and β_3_ in canine tumor extracts was not homogenous within the groups, and no relationship was found between integrin expression levels and tumor type, location or grade (Figure 5A). The expression of the α_V_ subunit appeared greater than the β_3_ subunit in mammary gland tumors and sarcomas, while the opposite was recorded for mast cell tumors.

Qualitative immunohistochemical analyses performed for integrin β_3_ also showed variable expression and heterogeneous localization of the subunit in the tumor tissues (Figure 5B). Mammary gland tumors showed moderate expression in both tumor cells and stroma (*i. e*., blood vessels, fibroblasts, myoepithelial cells), while mast cell tumors showed mainly stromal staining. Variable staining intensity was detected in the examined LNs, with predominant stromal localization. Semi-quantitative evaluation by the intensity scoring (0/3) system in the tumoral cells revealed a low-to-moderate integrin β_3_ expression in mammary tumors, a negligible expression in mast cell tumors and high expression in sarcomas (Table 5). Quantitative analysis of the total tumor stained area (tumor cells and stroma) revealed a moderate integrin β_3_ expression in mammary tumors, a low expression in mast cell tumors and high expression in sarcomas.

**Table 5:** Semi-quantitative (score) and quantitative (area) evaluation of anti-b_3_ integrin staining in tumor samples

Dog	Tumor type	IHC (b_3_ integrin in tumor cells, score 0/3)	IHC (b_3_ integrin positive area tumor area)
**2**	2a: Adenocarcinoma	1/3	25%
	2b: Adenocarcinoma	2/3	4%
**4**	4a: Adenocarcinoma	—	—
	4b: Adenoma	0.5/3	3%
**5**	5a: Leiomyosarcoma	3/3	53%
	5b: Adenoma	1/3	13%
**6**	Mast cell tumor	0/3	1%
**7**	Mast cell tumor	0/3	1%
**8**	Mast cell tumor	0/3	1%
**9**	Mast cell tumor	0/3	2%
**11**	Mast cell tumor	0/3	2%
**13**	13a: Adenocarcinoma	0.5/3	13%
**14**	Soft tissue sarcoma	2/3	13%
**15**	Osteosarcoma	3/3	48%
**16**	Melanoma	1/3	—
**17**	Adenocarcinoma	0.5-1/3	11%
**18**	Lipoma	0/3	1%
**19**	19a: Adenocarcinoma	2/3	16%
	19b: Adenoma	1/3	12%
**20**	20a: Adenocarcinoma	0.5/3	—
	20b: Adenocarcinoma	—	—
**21**	Adenocarcinoma	1-2/3	11%
**22**	Mast cell tumor	0/3	2%
**23**	Mast cell tumor	0/3	4%

Given the low sample size and the high variability in integrin expression levels among the samples, no statistical evaluations were performed.

## DISCUSSION

In this canine trial, preliminary safety and imaging efficacy of the fluorescent probe DA364 were assessed during fluorescence-guided surgery in dogs with spontaneous tumors, in combination with standard-of-care practice. The contrast agent was well tolerated after IV injection at all doses tested, and the median intra-operative TBR was > 1.8 for mammary tumors, mast cell tumors and sarcomas with TBRs up to 3.9, 5.6, and 4.3, respectively, reached in individual patients, suggesting overall good tumor delineation properties for DA364. Furthermore, DA364 pinpointed four wound beds that contained residual tumor cells (true positives), and 11 fluorescence-positive beds which were tumor-free, confirmed by histopathology (false positives). Overall, the results of the trial suggest that fluorescence-guided surgery using DA364 is safe and feasible, the contrast agent accumulates in different tumor types, and may help to detect residual disease in the wound bed. However, the exploratory nature of the trial and the different variables and protocols tested mandate to consider such results as preliminary; further investigations will be necessary to fully gauge the performance of DA364, particularly its diagnostic accuracy.

The TBRs varied greatly between and within tumor types, as also observed in recent veterinary trials assessing another integrin-targeting probe [24, 25]. Interestingly, the melanoma showed the lowest TBR, probably because the dark pigmented lesion strongly absorbed the excitation light. Qualitative intra-operative fluorescence examination revealed sharp tumor identification only for half of the lesions. The discrepancy between the semi-quantitative (TBR, post-processing) and the qualitative (subjective visualization, real-time) outcome may be related to several factors. First, qualitative intra-operative discrimination of lesions with a modest TBR (1.1–1.5) may be suboptimal compared to lesions with a higher TBR. Secondly, the hand-held device used in the trial carries an inherent position variability towards the surgical field, and therefore tissue signal intensity and relative contrast may slightly differ between intra-operative examination (e. g., multiple positioning during surgery) and post-processing analyses conducted on a single image. Automatic thresholding algorithms embedded in the imaging system software may facilitate future real-time tumor delineation based on the TBR. The use of fixed-distance intra-operative camera systems with automatic thresholding algorithms may also help to overcome these shortcomings in future trials.

Two previous veterinary clinical trials described the use of an integrin-targeting probe containing a cyclodecapeptide scaffold with 4 c (RGDfK) motifs and a fluorescent dye (Angiostamp, Fluoptics, Grenoble, France), during fluorescence-guided surgical resection of tumors in cats and/or dogs using the Fluobeam system [24, 25]. In the first trial, 12 cats with fibrosarcoma underwent fluorescence-guided surgery [24], whereas in a second study only one cat with mammary gland tumors and three dogs with different tumor types (mammary gland (2) and/or ovarian (2) disseminated tumors) were enrolled [25]. Angiostamp showed good accumulation in the tumors and high intra-operative TBRs. As pointed out by the investigators, the large tumor-free margin routinely taken in fibrosarcoma cases often emitted a moderate fluorescent signal due to peri-tumoral inflammation, potentially hampering distinct delineation of the tumor itself [24]. A direct comparison between canine and feline studies using Angiostamp for NIRF imaging and our trial using DA364 is challenging. Most important confounding factors include the different patient enrolment and tumor characteristics, the different spectral properties and dosing of the contrast agents, and technical variables related to the camera systems [26]. Another relevant parameter that may strongly affect the study outcome, particularly the TBR, is the definition of the ROIs. For the intra-operative images, we selected as “background” a region as close as possible to the primary tumor, either adjacent or even underneath when the tumor was flipped over. It is not clear how the background areas were selected in the Angiostamp studies to derive the TBR, and whether the ratiometric analysis was performed using an image containing both tumor and background ROIs (as in our study), or different images. A standardized method to select ROIs in fluorescence-guided surgery is necessary to perform an unbiased evaluation of the TBR [27]. The results of the Angiostamp studies and the present trial suggest that integrin-targeting approaches using small fluorescent contrast agents are capable to detect tumor lesions during surgical resection and to assist the surgeon beyond standard-of-care.

During the trial, different doses of the contrast agent were evaluated. The dose of 1.8 mg/m^2^ (0.05 mg/kg human estimated dose after allometric scaling) yielded the most optimal TBR for intra-operative detection of tumor lesions. At lower doses (0.06 – 0.6 mg/m^2^), the imaging performance of the contrast agent was suboptimal, probably due to low accumulation and low fluorescent signal detected by the camera system used in this trial which is known to underperform at low concentrations [26]. We believe that the use of a fluorescence camera with higher sensitivity may allow reduction of the drug dosage. Two imaging time-points (24 and 48 h PI) were evaluated to assess the relationship between background signal reduction and tumor contrast generation. Delaying the surgery till 48 h PI did not improve the TBR. After post hoc analysis of the signal decay, the T_1/2_ of DA364 retention in the tissues appeared to be 16.5 h PI. Therefore, future studies should investigate whether a decrease in time interval between injection and imaging would potentially result in improved *in situ* TBR and *ex vivo* TMR. Likely, the most appropriate protocol (dose and imaging time-point) will depend on the type of tumor, and future trials will be necessary to define the optimal protocol for a selected indication.

Besides intra-operative tumor identification, detection of residual disease is probably the other main unmet need that fluorescence-guided surgery may address, to reduce cancer recurrences due to positive margins, to prevent additional surgeries, and to improve the quality of life and survival of the patients. Indeed, the detection of residual disease is a primary endpoint for confirmatory human trials in fluorescence-guided surgery (*e. g*., NCT03180307 for ovarian cancer, NCT03686215 for breast cancer). NIRF imaging using DA364 highlighted four wound beds containing residual tumor lesions. Particularly in one dog in this trial, the surgeon considered the wound bed to be clean based on standard-of-care inspection, but fluorescence was noted in the caudal wound bed and histopathology confirmed positive margins. In this case, the use of DA364 may have prevented the dog a second surgery. The four true positive wound beds detected in this canine trial suggest positive performance of DA364 for residual disease identification during surgery. On the other hand, 11 wound beds emitting false positive fluorescence were detected, probably due to non-specific accumulation of DA364 in connective and lipid tissues, or binding of the fluorescent probe to the activated endothelial cells, present in the tumor and peri-tumoral environment [19]. In those dogs, additional biopsies were taken. Although no complications were associated with these additional resections, longer anaesthesia and surgery times were recorded. Of note, the vast majority of false positives tissues were removed in mast cell tumor and sarcoma cases, while only one false positive sample was obtained after mastectomy (in a dog receiving the highest tested dose). The rate of false and true positives is a very important parameter for a diagnostic agent, particularly when considering applications such as breast-conserving surgeries. For this trial, we documented the results case by case, listing all additional tissue samples that were removed after standard-of-care resection. At the same time, we would like to stress that these results should be considered preliminary, given the exploratory nature of the trial and the variety of variables tested (*i. e.,* different doses, different injection-to-imaging time-points, highly heterogeneous patient population and tumor characteristics). Further trials using DA364 will be necessary to assess the diagnostic accuracy of the agent in an optimal protocol and applied to a selected indication, using a fluorescence camera with high performance.

Fluorescence evaluation of resected specimen showed higher fluorescence signal in the center of lesions compared to the safety margin left by the surgeon, confirming the accumulation of DA364 in the lesion detected intra-operatively. *Ex vivo* ratiometric evaluation of the fluorescence in resected specimens revealed overall higher TMRs than intra-operative TBRs. This is probably because for *ex vivo* imaging, the specimen was cut in half before imaging, exposing deep fluorescent portions of the sample that may have been masked *in vivo* by overlying layers of tissue, and not readily detected by the NIRF camera which has limited penetration depth capabilities.

NIRF imaging using DA364 also showed preliminary efficacy for detection of metastasis in a small number of regional LNs. Intra-operative detection of a fluorescent LN might provide crucial additional information during surgery [28]. In this trial, the SBR of resected metastatic LNs was significantly higher than the SBR of non-metastatic LNs. However, *in situ* evaluation of LN fluorescence was challenging, probably due to the anatomical location (embedded in fat) of the nodes and the limited penetration depth of the technology [7]. Further evaluations will be necessary to confirm these preliminary findings on a larger population.

The presence of the integrin receptors was evaluated on resected specimen. Immunohistochemical evaluation of anti-β_3_ showed positive staining in sarcoma and mammary tumor cells, but not in mast tumor cells. Presence of the β_3_ receptor subunit was found in greater but variable extent in the tumor stroma, suggesting the presence of neoangiogenesis or integrin-expressing tumor-associated cells. In mast cell tumors, the expression of β_3_ is likely associated with the presence of tumor vascularization, since no staining was found in tumor cells. Western blot analyses conducted on punch biopsies revealed the presence of β_3_ protein in all tumor types, with heterogeneous expression. β_3_ protein is a reliable indicator of the α_v_β_3_ integrin receptor, since it only forms dimers with the α_V_ subunit on tumor epithelial and stromal cells (of note, α_IIb_β_3_ is only found on platelets), while a_V_ protein may also assemble with other β subunits. No apparent correlation was found between the presence of β_3_ integrin and DA364 signal in tumors. This is probably due to several factors, including the small amount of tissue submitted for target expression analyses (*i. e.*, punch biopsy and thin histological slice) which may not be representative for the whole tumor, but also the contribution of different mechanisms to the tumor accumulation properties of the contrast agent, such as passive diffusion through leaky vessels in addition to target-depended uptake. Furthermore, since the RGD-binding moiety can recognize different integrin receptor dimers other than α_v_β_3_, an evaluation conducted only on the β_3_ protein may be misleading and limitative to address the presence of the biomarkers pool potentially recognized by the molecular imaging agent. Here, we evaluated the expression of the α_V_ subunit by western blot as surrogate for the α_V_ integrin receptors pool. Similar to the variable presence of integrin subunit β_3_, a heterogeneous expression of the integrin α_V_ subunit was found in all tumor types. Nonetheless, we acknowledge that assessing the expression of the β_3_ and α_V_ subunits may be suboptimal, affected by the presence of cytoplasmic fractions not relevant for membrane targeted uptake, and not entirely representative of the final heterodimers population. Unfortunately, in this study the evaluation of the intact dimers was not possible by western blot because of the separation in monomers during tissue processing, and the lack of monoclonal antibodies for immunohistochemistry validated on canine tissue. This should not be a limiting factor for human translation since α_v_β_3_, α_v_β_5_ and α_V_β_6_ antibodies are available and validated for formalin-fixed paraffin-embedded human tissues [29].

We acknowledge some limitations of this study. First, a heterogenous patient population was enrolled with a relatively small number of cases within each tumor type. In veterinary medicine, it is common to pre-operatively assess the nature of the tumor, based on fine-needle-aspirates only. Cytology minimizes the cost for the owner and avoids anaesthesia, required for pre-operative core biopsies, but is less accurate than histology. Consequently, post-operative histopathology might identify another tumor type than expected based on pre-operative cytology, which was the case for some tumors enrolled in this trial. Second, the hand-held system used in the trial suffers from inherent positioning variability, field inhomogeneity and limited sensitivity. Some samples were not evaluated because of suboptimal image quality. Image quality would benefit from using a fixed-position camera with high sensitivity. Furthermore, the majority of tumors for which evaluation of the signal was not feasible were additional tumors that were diagnosed during physical examination and that had a small diameter (less than 5 mm). Measurement of the fluorescence in those samples appeared not feasible. Finally, no parallel control group (*e. g*., standard-of-care without fluorescence imaging) was included. A control arm may allow randomization and masking, with both arms receiving the contrast agent (to assess safety) and standard-of-care surgery, but only one cohort of patients submitted to fluorescence-guided surgery. This study design may allow to truly assess the added value of fluorescence-guided surgery by evaluating the number of fluorescent residual lesions identified in the wound bed, and the long-term effect on patient quality of life and survival.

## MATERIALS AND METHODS

### Contrast agent

The fluorescent contrast agent DA364 (Bracco Imaging SpA, Italy) was synthetized as previously reported [21]. The empirical formula of DA364 is C63H77N11O20S4 and the molecular mass is 1436.60 g/mol. DA364 has excitation/emission maxima at 676/696 nm and very high brightness (molar extinction coefficient: 275,000 L ^*^ mol^-1 *^ cm^-1^) in phosphate buffer saline (pH 7.4) (unpublished data).

### *In vitro* analyses

Receptor binding assay and cell uptake blocking experiments were performed as previously described [21]. A 96-well microtiter plate was coated overnight at 4° C with 1 μg/mL recombinant human α_v_β_3_ integrin (Merck-Millipore, Darmstadt, Germany) in coating buffer (20 mM Tris HCl pH 7.4; 150 mM NaCl; 1 mM MnCl_2_; 0.5 mM MgCl_2_; 2 mM CaCl_2_). After coating, the plate was rinsed in washing buffer, then incubated in blocking buffer (3% BSA in coating buffer) for 2 h, under shaking and at room temperature (RT). The plate was then rinsed in washing buffer and incubated with the testing compounds in the presence of 1 μg/mL biotinylated vitronectin (Molecular Innovation), for 3 h at RT. The plate was then rinsed again and incubated in streptavidin–HorseRadish Peroxidase solution (GE Healthcare, Chicago, USA) for 1 h at RT. After rinsing, the plate was incubated in TetraMethylBenzidine (Sigma-Aldrich, Darmstadt, Germany) solution for 5 min and the colorimetric reaction was stopped by adding 2 M sulfuric acid. The evaluation of the half-maximal inhibitory concentration (IC_50_) for the testing compounds was calculated by measuring the competitor (biotinylated vitronectin) by reading the absorbance at 450 nm (Optical Density). All samples were analyzed in triplicate and each assay was performed 3 times. IC_50_-values were determined by nonlinear regression analysis of the slope. For cell uptake experiments, WM266 human melanoma cells were seeded on 24-wells plate over 24 h, and then incubated with 1 μM of DA364 in fresh serum-free medium in presence or absence of unconjugated cRGD peptidomimetic (1, 5, 10, 50, 100 μM) for 20 min at 37° C. At the end of incubations, the cells were rinsed with cold PBS, collected and suspended in PBS. Cells were gated according to their light-scattering properties to exclude cell debris and cell fluorescence was analyzed (minimum 20000 events/sample) using Accuri C6 flow cytometer (Becton Dickinson, San Jose, USA). Excitation laser was set at 640 nm and the fluorescence emission was collected with 670 nm long pass filter. Three independent experiments were performed in triplicates. Results were expressed as percentage of the maximum uptake (*i. e*., cells incubated with DA364 in absence of unlabeled cRGD).

### Patient enrollment

Twenty-four client-owned dogs with a tentative diagnosis of surgically manageable solid neoplasia based on localization and palpation (mammary gland tumor) or cytology (melanoma, (sub) cutaneous mast cell tumor, (adeno) carcinoma or sarcoma) were considered for enrolment in the trial. The local research ethical committee granted ethical approval for this clinical trial (Institutional Animal Care and Use Committee; EC2015/124). Additionally, this project received approval of the deontological committee of the Federal Public Service Health, Food Chain Safety and Environment for the enrolment of non-purpose-bred dogs. All owners signed an informed consent form. Pre-surgical staging according to the World Health Organization (WHO) guidelines was completed the day of diagnosis; a thorough physical examination, complete routine blood analysis (hematology and biochemistry), urinalysis, thoracic radiographs (3 orthogonal projections), and/or abdominal ultrasound with guided fine-needle aspiration of regional superficial LNs (if applicable) were performed. Dogs were eligible when no distant metastases were detected and when there were no indications for kidney and/or hepatic impairment based on blood and urinalysis and medical imaging.

### DA364 administration

Prior to administration of the contrast agent, heart rate, respiratory rate, and systolic blood pressure were determined. In dogs diagnosed with a mast cell tumor, 0.2 mg/kg promethazine (Phenergan, Hikma, Hoorn, The Netherlands) was administered intramuscularly (IM) to prevent potential histamine release. Dogs received either 0.06 mg/m^2^, 0.6 mg/m^2^, 1.8 mg/m^2,^ or 3 mg/m^2^ as an IV bolus of undiluted DA364 via a cephalic vein (Table 1). The dose of DA364 was calculated based on the Body Surface Area. Before and after the administration of the contrast agent, the catheter was flushed with 2 mL sodium chloride (NaCl 0.9%, B. Braun Melsungen AF, Germany) to ensure patency, and then a mandarin was inserted until anesthetic induction at the time of surgery.

### Fluorescence imaging

Near-infrared fluorescence imaging was performed using the clinical Fluobeam 700^®^ (Fluoptics, Grenoble, France), a commercially available hand-held system which consists of a control unit with a laser source emitting at 680 nm, apart from an optical head with a charge-coupled device camera and white light emitting diodes for the illumination of the field of view, equipped with a long-pass emission filter (>700 nm). The optical head was fixed in a custom-made tripod, ensuring a fixed working distance of 17 cm to reach a 6-cm spot diameter at the field of view during pre- and postoperative imaging. For intra-operative imaging, the optical head was hand held and a similar working distance was pursued.

### Pre-operative imaging

The probe was administered 24 or 48 h prior to surgery. Transcutaneous imaging was performed 5, 10, 30 and 60 min PI and then hourly until 6 h and 24 h PI in dogs that received the probe 24 h prior to surgery. Transcutaneous imaging was performed 5, 10, 30, 60 min, 24 h and 48 h PI in dogs that received the probe 48 h prior to surgery. Dogs were hospitalized and vital parameters (heart rate, respiratory rate and systolic blood pressure) were assessed at predetermined intervals to screen for adverse effects until surgery. Acquired images were analyzed using a commercial image analysis tool (ImageJ v1.52). Regions of interest (ROIs) were drawn on tumor tissues based on the location of the mass identified during visual inspection and palpation, and the signal obtained was used to assess the tissue kinetics of the contrast agent.

### Intra-operative imaging

The Fluobeam 700^®^ was covered with a dedicated single-use sterile cover. In dogs diagnosed with a mast cell tumor, administration of promethazine was repeated immediately prior to anaesthetic induction. The choice of general anesthesia and analgesia protocols was based on the attending anesthesiologist’s preference. Surgical preparation was routinely performed. Surgical approaches were identical to those routinely used in dogs under white light with routine surgical margins and *en bloc* regional LN resection (if applicable). The surgery was briefly interrupted at several occasions to acquire images and videos while the operation and ambient lights were turned down. Images of the surgical field were obtained before the surgical incision and once the tumor was partially detached from the surrounding tissue and folded over so that the deep border could be imaged *in situ* at the same time as the wound bed. After removal of the tumor, the wound bed was evaluated by NIRF imaging to assess if there was residual fluorescent signal. Remaining fluorescent tissue was removed or, if this fluorescent tissue was outsized or removal deemed delicate, a biopsy was taken; those samples were identified as “fluorescence positive” and processed separately. A small tissue sample of tumor tissue was immediately frozen at –80° C for western blot analysis. When all images and samples were obtained, the surgical wound was closed routinely and the dog was recovered from anesthesia. Images were analyzed using a commercial image analysis tool (ImageJ v1.52) to assess the fluorescent signal in tumor and background tissue. The background tissue was defined as a putative healthy tissue close to the tumor, either the wound bed or an adjacent tissue in case tumor and bed were not in the same image. The fluorescence signal intensity from the tumor and background tissue was obtained by drawing ROIs on the tissue flap during removal, and the ratio was used to calculate the TBR (Figure 3).

### Post-operative imaging

Images of the excised tissue samples were acquired immediately after surgical removal of the tumor. Tumor and surrounding tissue, fluorescence-positive samples of the wound bed, and the LNs (if applicable) were imaged on a black surface. The tumor itself was then cut in half along its longitudinal axis and imaged with the cut planes facing upwards. Regions of interest were drawn on tumor and safety margins on the acquired *ex vivo* images using a commercial image analysis tool (ImageJ v1.52), and the fluorescent signal obtained was used to calculate the TMR. Regions of interest were also drawn on the resected LNs and adjacent fat tissue to derive the SBR. All samples were then fixed in abundant amounts of 10% formalin (CellStor Pot, CellPath, Wales, United Kingdom) for 24 h. Subsequently, half the tumor was further divided in slices of 3-5 mm thickness. A slice (tumor and surroundings) with maximal fluorescence was trimmed to fit into a histology cassette (UniSette, Sacramento, US), the most fluorescent side facing down, and re-immersed in a formalin container.

### Western blot

Tissue samples were homogenized by Precellys homogenizer directly in Lysis buffer (50 mM Tris-HCl pH 8; 150 mM NaCl; 1 mM EDTA; 100 mM NaF; 10% glycerol; 1 mM MgCl_2_; 1% TritonX-100; Protease Inhibitors), and put in ice for 20 min. Some samples, particularly those difficult to homogenize, were subsequently pulverized using mortar and pestle in presence of liquid nitrogen. The final extract was sonicated for 15 sec and centrifuged at 14000 g at 4° C for 15 min. The protein content of the supernatant was quantified by the bicinchoninic acid assay method: 50 μg of the total extract of each sample was loaded in a 10% polyacrylamide gel and electrophoresis was performed to separate the proteins by their molecular weight in denaturing conditions (20% Sodium Dodecyl Sulfate). Proteins were then transferred onto a Polyvinylidene difluoride membrane and subsequently blotted with the antibodies anti-integrin subunit β_3_ (#BK13166, Cell Signaling, Leiden, Netherlands) and anti-integrin subunit α_V_ (#Ab179475, Abcam, Cambridge, United Kingdom) to evaluate protein expression, and with the antibody anti-actin (#BK4970, Cell Signaling, Leiden, Netherlands), to allow signal normalization on the loaded proteins. Specific signal was visualized as a single or multiple band by an electrochemiluminescence detection method. The images of the blotted membrane were acquired by a ChemiDoc MP Imaging system and the area of the bands relative to the β_3_ expression levels were quantified with the software Image Lab v.4.1. The western blot analysis was repeated at least three times for all dog samples.

### Histopathology

All formalin-fixed tissues were further trimmed, placed in cassettes and subjected to standard dehydration procedure followed by embedding in paraffin (60–65° C). Four μm-thick slices were cut from the paraffin blocks by a microtome. A representative slice of each block was routinely stained using hematoxylin and eosin. Histopathological evaluation was executed by a board-certified veterinary pathologist. Fluorescence-positive tissue samples of the wound bed and the LNs (if applicable) were screened for the presence of tumor cells. The guidelines that the board-certified pathologist used to evaluate the tumor margins are based on the published guidelines by Kamstock and colleagues [30]. Samples containing (sub) cutaneous tumours (mast cell tumours) or sarcomas underwent cross-sectioning. In case of mammary gland tumours, each mammary gland was sampled as well as the lateral margins and the regional LNs. (Oral) melanomas also underwent cross-sectioning.

### Immunohistochemistry

Tissue immunostaining was performed using an anti-β_3_ integrin antibody (monoclonal rabbit anti-integrin β_3_ antibody, clone EPR2417Y, #ab75872, Abcam, Cambridge, United Kingdom) and, as control, an anti-IgG antibody with matching concentration (rabbit IgG isotype control antibodies, #3900, Cell signaling, Leiden, Netherlands). Serial sections were stained using routine hematoxylin and eosin for morphology reference. The expression of β_3_ integrin in tumor cells and stromal tissues was assessed via qualitative, semi-quantitative and quantitative evaluations. Qualitative evaluation was done based on the description of the localization of the labelling (tissue, structures, cell types). The expression of β_3_ integrin in tumor cells was semi-quantified with a 4-tiered score (0: rare or none; 1: <30% of positive tumors cells; 2: 30% to 65% of positive tumor cells; 3: >65% of positive tumor cells). This visual evaluation assessed the labelling in the tumor cells only (stroma not included). A quantitative evaluation of the surface with IHC staining in selected tumor areas was achieved using HaloTM software (IndicaLabs, Albuquerque, NM). The selected tumor regions were chosen as large as possible, avoiding areas with artifacts (tissue folds, non-specific staining). The results were expressed as a percentage of stained areas per selected tumor surface. This represented an evaluation of the labelling in the tumor cells and in the stroma.

### Statistics

Normality was confirmed for the *in vivo* TBR obtained in benign and malignant tumors, and *ex vivo* STB obtained in removed LNs (Kolmogorov-Smirnov test and Shapiro-Wilk test). Statistical differences between the TBR of malignant versus benign tumors, the TBR of malignant tumors in patients that received the product at 24 h vs 48 h prior to surgery, and STBs in normal versus metastatic LNs were evaluated using the independent *t*-test. A *p*-value < 0.05 was considered significant. Normality was not confirmed for the *ex vivo* TMR values in benign and malignant tumors nor for the individual tumor types (mammary tumors, mast cell tumors and sarcomas). Statistical differences between the TMR of malignant versus benign tumors were evaluated using the Mann–Whitney *U* test. A *p*-value of < 0.05 was considered significant.

## CONCLUSIONS

The integrin-targeting fluorescent probe DA364 did not raise safety concerns and showed accumulation in different types of spontaneous tumors. Fluorescence-guided surgery using DA364 is feasible, and may facilitate the delineation of primary lesions and the identification of residual disease missed by standard-of-care inspection. Larger clinical trials based a uniform patient population, a sensitive imaging system, and a specific dosing and imaging protocol are necessary to allow evaluation of sensitivity and specificity of the novel contrast agent.

## Abbreviations

ICG: Indocyanine Green
IM: intramuscularly
IV: intravenous
LN: lymph node
NIRF: near-infrared fluorescence
PI: post-injection
RGD: Arg-Gly-Asp
ROI: region of interest
RT: room temperature
TBR: tumor-to-background ratio
TMR: tumor-to-margin ratio
SBR: signal-to-background ratio
WHO: world health organization
